# Speed of Sound Measurements of Binary Mixtures of *trans*-1,2-Difluoroethylene (R-1132(E)) with Difluoromethane (R-32) or 2,3,3,3-Tetrafluoropropene (R-1234yf)

**DOI:** 10.1021/acs.jced.4c00531

**Published:** 2025-01-28

**Authors:** Aaron J. Rowane, Elizabeth G. Rasmussen

**Affiliations:** Applied Chemicals and Materials Division, National Institute of Standards and Technology, Boulder, Colorado 80305, United States

## Abstract

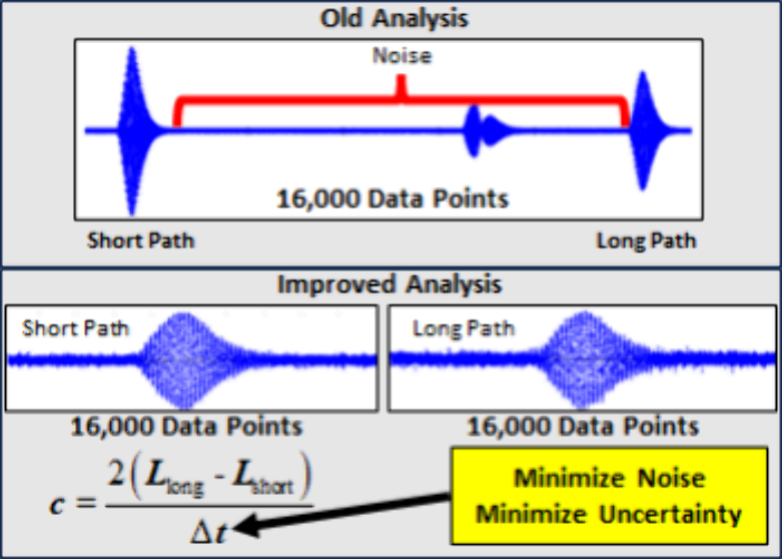

The speed of sound of two binary mixtures containing 0.336 mole fraction *trans*-1,2-difluoroethylene (R-1132(E)) with difluoromethane (R-32) and 0.435 mole fraction R-1132(E) with 2,3,3,3-tetrafluoropropene (R-1234yf) was measured with a dual-path pulse-echo instrument. The speed of sound was measured along several pseudoisochores for each blend at temperatures ranging from 230 to 325 K for blends with R-32 and to 342 K for blends with R-1234yf. Measurements started at pressures just above each mixture’s bubble point pressure and were limited to 8 MPa to avoid potential disproportionation reactions of R-1132(E). The data were compared to multifluid models incorporating Helmholtz-energy-explicit equations of state (EOS) for each pure fluid. No binary interaction parameters for either the R-1132(E)/32 or R-1132(E)/1234yf system are currently available. Therefore, binary interaction parameters for chemically similar systems suggested by REFPROP version 10.0 were used. Deviations from the measured data to the EOS ranged from 5.5 to 11.0% for the R-1132(E)/32 system and from 0.4 to 1.8% for the R-1132(E)/1234yf system. These data will be used to refit the R-1132(E) EOS and fit mixture models for R-1132(E) blends with R-32 and R-1234yf.

## 1 Introduction

The hydrofluoroolefin (HFO) *trans-*1,2-difluoroethylene (R-1132(E); C_2_H_2_F_2_, CAS#: 1630-78-0) is a low-global-warming-potential (GWP) refrigerant that was identified by McLinden et al.^1^ as one of six novel molecules for which very little thermodynamic data are available. Simulations by McLinden et al. showed that R-1132(E) has a greater coefficient of performance (COP) relative to the widely used hydrofluorocarbon (HFC) blend R-410A but a lower volumetric refrigeration capacity. The simulation also showed that of the HFOs identified by McLinden et al., R-1132(E) was found to have the highest COP. Some additional advantages of R-1132(E) are that it has zero ozone depletion potential and a GWP on a 100 year time frame of 1. R-1132(E) has a critical temperature, pressure, and density of 348.82 K, 5.172 MPa, and 438 kg·m^–3^, respectively.^2^ R-1132(E) is also a component of the refrigerant blend R-474A, which is being considered for use in automotive heating and cooling applications.^3^ The composition of R-474A is 23 mass % R-1132(E) with the balance made up of 2,3,3,3-tetrafluoropropene (R-1234yf). As highlighted by McLinden et al.^1^ and Giménez-Prades et al.,^4^ very little knowledge of the thermodynamic properties of R-1132(E) is presently available, which makes determining its true potential as a refrigerant challenging.

The R-1132(E) thermodynamic data available in the literature include vapor pressure data and critical properties reported by Perera et al.,^2^ saturated liquid densities by Utupala and Perera,^5^ surface tension data reported by Imai et al.,^6^ and compressed liquid densities by Sakoda et al.^7^ Sakoda et al. also reported saturated liquid densities and critical parameters. In addition to the thermodynamic properties of R-1132(E), its stability was investigated by Goto et al.,^8^ who found that R-1132(E) undergoes a pressure-driven disproportionation reaction at pressures exceeding 1 MPa in the presence of an ignition source. This disproportionation reaction was found to be mostly independent of the temperature. The same experiments were performed on mixtures of R-1132(E) with R-1234yf. Goto et al. found that by maintaining the concentration of R-1132(E) below 0.47 mole fraction in a mixture with R-1234yf, the disproportionation reaction could be suppressed. Compressed liquid thermodynamic properties over a wide range of state points are needed to develop a Helmholtz-energy-explicit EOS capable of reproducing reference quality thermodynamic properties. Accomplishing this goal requires measurements beyond a pressure of 1 MPa. Pulse echo experiments require the excitation of a transducer that could potentially serve as an ignition source for R-1132(E). Therefore, rather than attempt measurements with the potentially unstable R-1132(E), we performed measurements on two blends of R-1132(E) with difluoromethane (R-32) and R-1234yf. These measurements were performed with the intent to extract a Helmholtz-energy-explicit EOS for R-1132(E) from multifluid models of R-1132(E) blends with R-32 and R-1234yf.

Akasaka and Lemmon^9^ report a Helmholtz-energy-explicit equation of state (EOS) for R-1132(E), which is documented as having an uncertainty of 0.15% in vapor pressure, 0.1% in liquid densities, 0.4% in vapor densities, and 0.1% in vapor-phase sound speeds. Deviations in density are as high as 2.0% in the critical region. No uncertainty is assigned to the speed of sound in the compressed liquid state given the lack of available data. Extracting a Helmholtz-energy-explicit EOS for R-1132(E) from multifluid models of R-1132(E) blends with R-32 and R-1234yf requires vapor–liquid equilibrium, density, and speed of sound data for the blends at a variety of state points. However, the scarcity of data for blends of R-1132(E) with other refrigerants is even more pronounced than that for pure R-1132(E). Presently, only a single density data set, reported by Utupala and Perera,^5^ is publicly available for a 50/50 mass % blend of R-1132(E) with R-1234yf. No literature data are available for R-1132(E)/32 blends. In this study, we report the speed of sound data for blends of 0.336 mole fraction R-1132(E) with the balance being R-32 and 0.435 mole fraction R-1132(E) with the balance being R-1234yf at temperatures ranging from 230 to 342 K and pressures up to 8 MPa. The data are compared to multifluid models incorporating the latest Helmholtz-energy-explicit EOS for R-1132(E),^9^ R-32,^10^ and R-1234yf^11^ and binary interaction parameters of chemically similar systems suggested by REFPROP version 10.0.^12−14^

## 2 Materials and Methods

The features of the dual-path pulse echo instrument are presented by McLinden and Perkins in detail^15^ and are only briefly summarized here. Unique to this study are the sample preparation and an updated data analysis procedure, which are discussed in detail.

### 2.1 Sample Preparation

The mixture samples were received directly from the manufacturer in the liquid phase. Table 1 lists general information about the pure refrigerants including their short names, molar mass, and source. The supplier did not provide sample purity information; therefore, the presence of components other than R-1132(E), R-32, and R-1234yf was determined from NMR. The impurity in the mixture samples was found to be 0.0065 mole fraction for the R-1132(E)/32 blend and 0.0067 mole fraction for the R-1132(E)/1234yf blend.

**Table 1:** Refrigerants Present in the Mixtures Used in This Study Are Listed with Their CAS Numbers, Molar Mass, and Source

chemical name	CAS number	molar mass (g·mol^–1^)	source
*trans*-1,2-difluoroethylene (R-1132(E))	1630-78-0	64.0	Daikin
fifluoromethane (R-32)	75-10-5	52.02	Daikin
2,3,3,3-tetrafluoropropene (R-1234yf)	754-12-1	114.04	Daikin

Liquid-phase mixtures of R-32 or R-1234yf with less than 0.47 mole fraction of R-1132(E) were obtained directly from the manufacturer. These mixture samples were intended to be used to charge several instruments with the sample. Charging a liquid sample into each instrument would have resulted in a shift in the sample’s bulk composition between loadings of each instrument. To avoid this fractionation between loadings, the entirety of the liquid-phase samples received from the manufacturer was expanded into larger cylinders to store the samples in the vapor phase. The compositions provided by the manufacturer were approximate. Therefore, the compositions for each mixture were determined from nuclear magnetic resonance (NMR) using the procedure outlined by Suiter et al.^16^Table 2 lists the compositions of each mixture determined using NMR with their combined standard uncertainties.

**Table 2:** Mixture Compositions for the Studied Binary Mixtures Are Listed with Their Combined Standard Mole Fraction Uncertainties^a^

mixture	*x*_1,NMR_ /mole frac.	*u*_c_(*x*_1,NMR_)/mole frac.
R-1132(E)/1234yf	0.435	0.004
R-1132(E)/32	0.336	0.003

a The component 1 mole fractions listed are for R-1132(E).

### 2.2 Dual-Path Pulse-Echo Instrument and Data Analysis

The speed of sound was measured using the same dual-path pulse-echo instrument described in our previous studies.^15,17,18^ The instrument is capable of measurements of liquids from 228 to 423 K and pressures up to 93 MPa. In this study, measurements were terminated at 325 K for the R-1132(E)/32 blend and 342 K for the R-1132(E)/1234yf blend. The pressure was limited to 8 MPa to avoid a potential pressure-driven disproportionation reaction of the R-1132(E). The standard uncertainties in the temperature and pressure were 0.005 K and 0.014 MPa, respectively.

**Figure 1:**
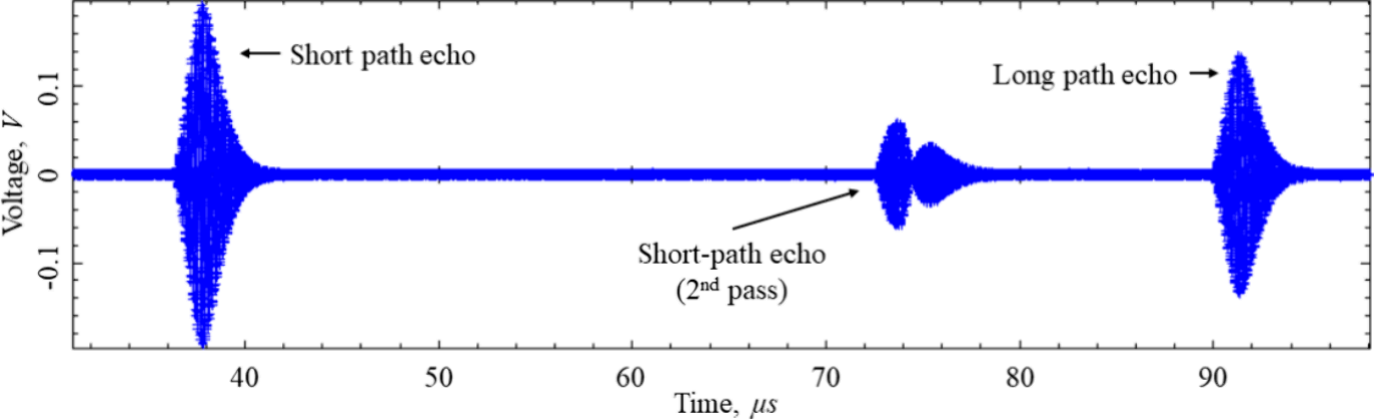
Oscilloscope trace used to obtain Δ*t* in previous studies^15,17−20^ encompassing 16,000 points inclusive of the first and second pass of the short path echo and long path echo. Reproduced from ref (17). Copyright 2022 American Chemical Society.

While the details of the instrumentation were the same as our previous studies, there is one key difference in the data analysis procedure. The speed of sound is determined by
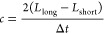
1where *L*_long_ and *L*_short_ are the length of the long and short paths, respectively, by which the sound burst emitted from the pulse echo’s transducer travel, and Δ*t* is the time difference between the return of the long and short patch echoes to the transducer. Figure 1 shows an example of echo data used in previous studies^15,17−20^ to determine Δ*t,* which includes both the short and long path echo data in addition to noise between the echoes that is not useful for determining Δ*t*. The oscilloscope used in this work had a maximum storage capacity of 16,000 data points, so as Δ*t* increased, the short and long path echoes' resolution decreased, ultimately increasing the uncertainty in the speed of sound measurement. As noted by McLinden and Perkins,^15^ an appreciable increase in the speed of sound uncertainty occurs at sound speeds lower than 600 m·s^–1^. McLinden and Perkins modified the instrument control code to independently capture higher-resolution short and long path echoes to minimize the uncertainty in Δ*t.* While independent short and long path echo data were captured in previous studies,^15,17−20^ they were not used to analyze the speed of sound data. In this study, the data analysis code was modified to utilize the independent short and long path echo data to determine Δ*t*. The procedure used to determine Δ*t* from the echo data is the same as that described by Ball and Trusler,^21^ and the interested reader is referred to that study for a detailed description.

**Figure 2:**
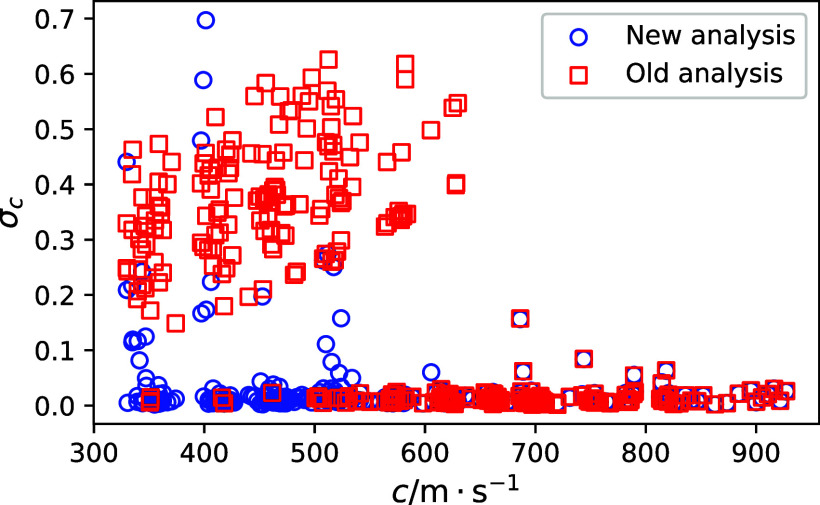
Standard deviation in the measured speed of sound, σ_*c*_, versus the measured speed of sound, *c*, when determining Δ*t* with independently recorded short and long path echo data (new analysis) and when determining Δ*t* with echo data inclusive of the short and long path echoes like that shown in Figure 1 (old analysis).

**Figure 3:**
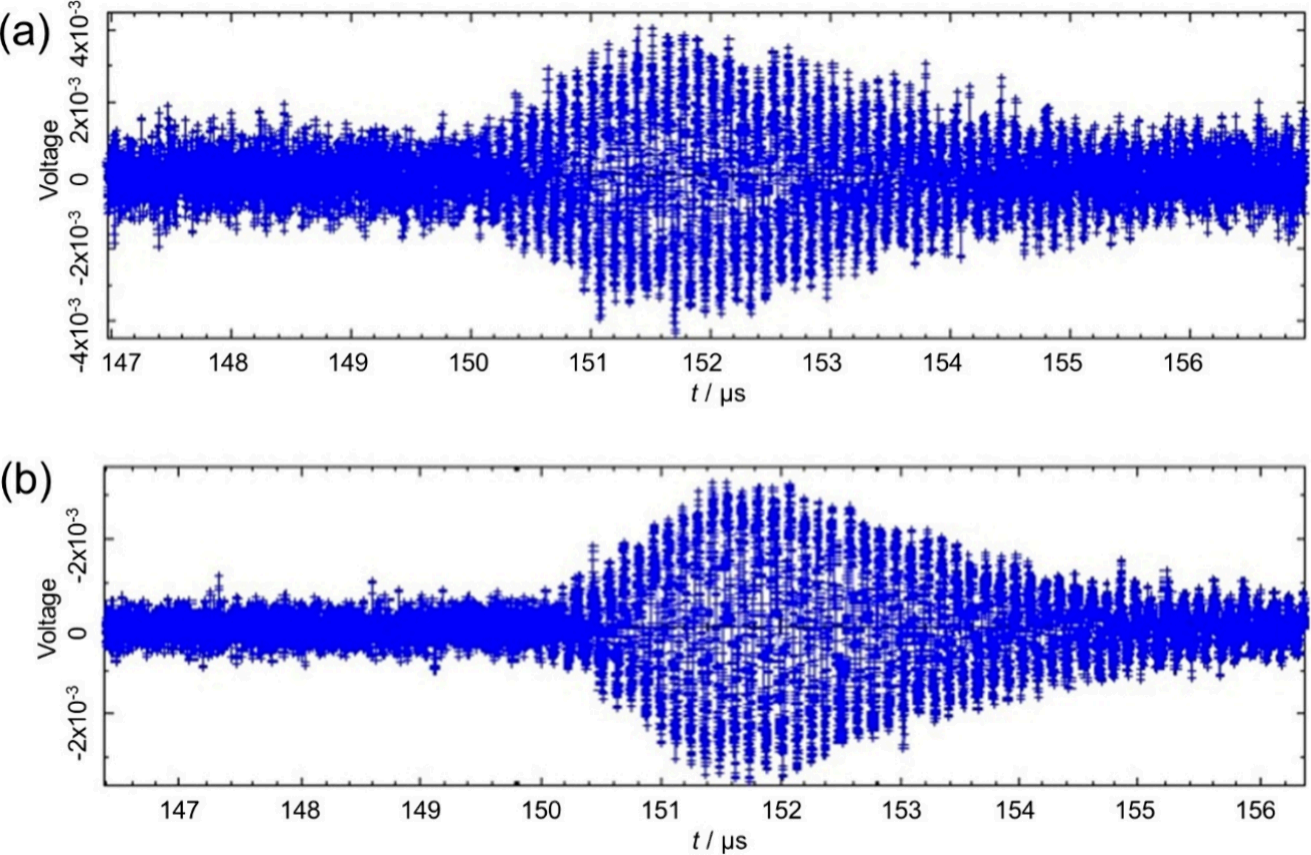
Long path echo data at 310 K and 1.7 MPa for the R-1132(E)/1234yf mixture when (a) 256 and (b) 1024 echoes are averaged. At this state point, the measured speed of sound was 397.34 m·s^–1^.

The uncertainty contribution from Δ*t* can be ascertained from the standard deviation of the 12 replicate speed of sound measurements at each (*p*, *T*) state point. Figure 2 shows the standard deviation in the speed of sound versus the speed of sound when obtaining Δ*t* from independently recorded echo data and when obtaining Δ*t* from echo data inclusive of the short and long path as shown in Figure 1. Figure 2 shows a large increase in the speed of sound standard deviation at speed of sound values below about 600 m·s^–1^ when using echo data inclusive of the short and long path. In a few select cases, larger standard deviations of up to 0.7 m·s^–1^ occurred when independent echo data were used to determine Δ*t*. This was not due to an insufficient number of echo data points but was a result of insufficient averaging of the echo data. Typically, we have averaged 256 echoes, which usually provide a sufficiently high signal-to-noise ratio for accurate speed of sound measurements. However, for a few select isochores, it was necessary to increase the averaging up to 1024 echoes to increase the signal-to-noise ratio on the long path echoes to obtain a low standard deviation in the measured speed of sound. Figure 3 compares the long path echoes with an averaging of 256 versus 1024 echoes at a temperature of approximately 310 K and a pressure of 1.7 MPa. This increased averaging decreased the standard deviation in the speed of sound by a factor of 3. Through visual examination of the two echoes, one can see that the long path echo with the increased averaging appears more defined.

The relative expanded speed of sound uncertainties, *U*_r_(*c*), were determined with a coverage factor, *k* = 2, and included effects of composition, temperature, pressure, time delay between echo arrivals, and path length calibration. More details on the uncertainty analysis can be found in our previous studies.^15,17^ Minimizing the uncertainty in Δ*t* by the analysis of the independent echoes resulted in the largest contributions to the uncertainty in the speed of sound to be that of the pressure and composition. The rate of change in the speed of sound with pressure increased as the system approached the sample mixture critical point. The estimated mixture critical temperatures for the R-1132(E) blends with R-32 and R-1234yf were 344.97 and 359.58 K, respectively, which were calculated using the mixture models implemented in REFPROP version 10.0 that are discussed in Section 3. The composition uncertainties listed in Table 2 are an order of magnitude greater than those achievable with gravimetric sample preparation and added 0.04 to 0.06% to the relative expanded uncertainty in the speed of sound measurement. In this study, the lowest achievable relative expanded uncertainty in the speed of sound was 0.08%, in contrast to those in previous studies that were less than 0.04%. This contribution is more than a 2-fold increase in the relative expanded uncertainty in the speed of sound.

### 2.3 Sample Loading

Much of the details of the loading procedure remain the same as those presented in our previous studies^17,19,20^ where vapor mixture samples were prepared and loaded into the measuring cell. Prior to loading any sample, the instrument’s filling lines, manifold, and measuring cell were evacuated to an approximate pressure of 8 × 10^–4^ Pa for 12 h. The measuring cell was then cooled to 228 K. Once the temperature of the measuring cell had stabilized, the internal volumes were closed off to vacuum, and the sample cylinder valve was opened; the sample began flowing through the lines of the manifold and condensed into the chilled portions of the filling lines located in the thermostat bath and the measuring cell. It is important to note that the sample cylinder pressure far exceeded that of either mixture’s dew point pressure at 228 K. As discussed in our previous studies,^17,19^ initially, there is a possibility that sample fractionation could occur as the system pressure approached the cylinder pressure. However, once the system approached the cylinder pressure, the sample was forced to condense at the prepared composition. As the measuring cell began to fill with liquid, the sample fraction that condensed first was pushed out of the cell into part of the manifold, which are volumes that do not impact the measurement. Measurements commenced once the measuring cell was filled with the liquid.

### 2.4 Isochoric Measurement Procedure

Speed of sound measurements using the dual-path pulse-echo instrument were performed along pseudoisochores. The internal volumes of the instrument are nearly fixed in volume, changing only with the temperature and pressure from the effects of thermal expansion and compressibility of the component’s materials. The state point of the sample was varied by changing the temperature. Immediately following the loading, vapor still resided in the instrument manifold, resulting in small changes in pressure when varying the measuring cell’s temperature. We call this initial phase of the measurements the “saturation trace” where the measured pressure corresponds to a bubble point pressure intermediate to the cell temperature and room temperature (296 K).

As the temperature of the measuring cell increased, the liquid sample expanded and filled the instrument manifold, resulting in larger increases of pressure when varying the temperature. Measurements were made starting at two different loading temperatures (230 and 260 K) for both mixtures. The temperature increment between state points was variable, ranging from 1 K at the lowest temperatures where the sample was most incompressible up to 2 K at the higher temperatures where the sample was more compressible. Once the system pressure reached 8 MPa along a pseudoisochore, the measuring cell was cooled to the next starting temperature, and the sample was removed from the system to reduce the pressure to approximately 0.5 MPa above the bubble point pressure estimated using mixture models implemented in REFPROP version 10.0.^12^ This process was repeated until the echo signals were too weak to obtain speed of sound data with low uncertainties, which occurred at temperatures of 325 K for the R-1132(E)/32 blend and 342 K for the R-1132(E)/1234yf blend.

## 3 Results and Discussion

The following sections present the speed of sound data measured using the dual-path pulse echo instrument for the R-1132(E)/32 and R-1132(E)/1234yf blends. The data are compared to multifluid models for both mixtures implemented in REFPROP version 10.0.^12^

### 3.1 Experimental Speed of Sound Data

**Figure 4:**
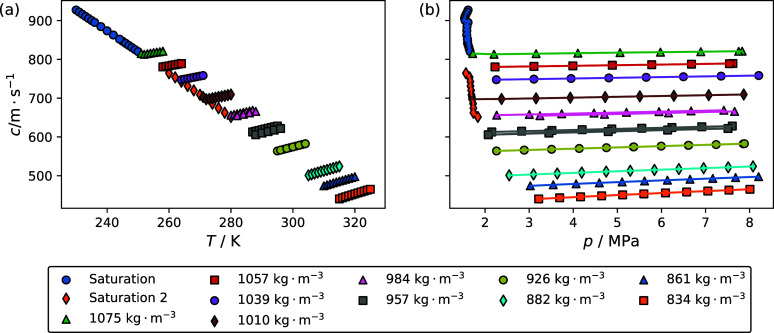
Impact of (a) temperature and (b) pressure on the speed of sound for the blend with a composition of 0.336 mole fraction R1132(E) and 0.664 mole fraction R-32. Symbols listed in the legend are for each pseudoisochore for which measurements were performed. Lines in panel b are drawn to guide the eye. Saturation 1 and saturation 2 correspond to data that were taken while the sample manifold was not filled with liquid for the 230 and 260 K sample loadings, respectively.

**Figure 5:**
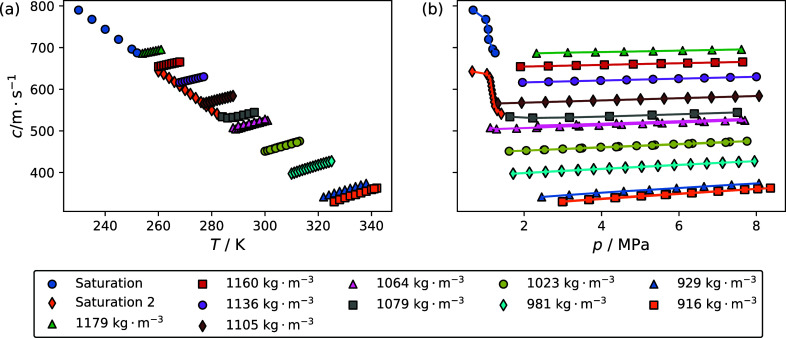
Impact of (a) temperature and (b) pressure on the speed of sound for the blend with a composition of 0.435 mole fraction R1132(E) and 0.565 mole fraction R-1234yf. Symbols listed in the legend are for each pseudoisochore for which measurements were performed. Lines in panel b are drawn to guide the eye. Saturation 1 and saturation 2 correspond to data that were taken while the sample manifold was not filled with liquid for the 230 and 260 K sample loadings, respectively.

Figure 4a,b shows the impact of the temperature and pressure on the speed of sound of the R-1132(E)/32 blend. Different symbols represent different pseudoisochores for which the corresponding density was estimated from a multifluid model incorporating the Helmholtz-energy-explicit EOS reported by Akasaka and Lemmon^9^ for R-1132(E) and Tillner-Roth and Yokozeki^10^ for R-32 with binary interaction parameters for the chemically similar R-32/1234yf system.^13^Figure 5a,b shows the impact of temperature and pressure, respectively, on the speed of sound of the R-1132(E)/1234yf blend. The densities for each pseudoisochore were estimated using a multifluid model incorporating EOS reported Akasaka and Lemmon for R-1132(E) and Lemmon and Akasaka for R-1234yf.^11^ Both Figures 4 and 5 show several repeat isochores. Tables 3 and 4 list experimental speed of sound data along with the corresponding temperature, pressure, and relative expanded state point uncertainty (*U*_r_(*c*)) for the R-1132(E)/32 and R-1132(E)/1234yf blends, respectively. Data listing all of the unaveraged speed of sound measurements and their associated uncertainties can be found in the Supporting Information, and these data are also deposited at nist.data.gov (DOI: https://doi.org/10.18434/mds2–3565).

**Table 3:** Experimental Speed of Sound Data for the R-1132(E)/32 Mixture with a Composition of 0.336 Mole Fraction R-1132(E) and 0.664 Mole Fraction R-32 (*c*) Listed with the Corresponding Temperature (*T*), Pressure (*p*), and Relative Expanded Uncertainty (*U*_r_(*c*)) Determined with a Coverage Factor, *k* = 2^a^

*T*/K	*p*/MPa	*c*/m·s^–1^	100·*U*_r_*(c)*
229.999	1.616	927.34	0.08
231.004	1.596	921.89	0.08
232.010	1.569	916.36	0.08
233.016	1.546	910.86	0.08
234.010	1.534	905.47	0.08
235.009	1.547	900.20	0.08
235.992	1.615	895.33	0.09
238.000	1.591	884.45	0.08
240.002	1.586	873.67	0.09
242.012	1.585	862.84	0.09
244.001	1.601	852.19	0.09
245.005	1.605	846.79	0.09
245.996	1.604	841.40	0.09
247.008	1.619	836.00	0.09
248.006	1.634	830.68	0.09
249.017	1.646	825.27	0.09
250.003	1.663	820.02	0.09
251.006	1.719	814.94	0.09
251.994	2.204	813.17	0.09
252.998	3.158	814.60	0.09
253.996	4.105	816.03	0.09
255.014	5.062	817.43	0.09
256.017	5.978	818.60	0.09
257.033	6.893	819.73	0.08
258.020	7.831	821.16	0.08
258.021	7.751	820.66	0.08
258.005	2.226	780.51	0.10
258.991	3.109	781.94	0.10
259.979	3.990	783.35	0.09
260.983	4.886	784.78	0.09
261.982	5.779	786.22	0.09
262.983	6.680	787.71	0.09
263.985	7.622	789.47	0.09
263.985	7.542	788.90	0.09
259.992	1.567	764.18	0.10
261.980	1.651	753.71	0.10
263.998	1.667	742.48	0.11
265.995	1.670	731.21	0.11
267.993	1.686	719.96	0.11
269.987	1.692	708.60	0.11
271.994	1.715	697.23	0.12
273.995	1.729	685.72	0.12
275.980	1.736	674.17	0.12
277.978	1.736	662.38	0.12
264.001	2.256	747.54	0.10
265.004	3.109	749.08	0.10
265.995	3.951	750.59	0.10
267.000	4.807	752.13	0.10
267.992	5.654	753.68	0.10
268.983	6.498	755.20	0.09
269.985	7.352	756.75	0.09
270.986	8.206	758.29	0.09
270.985	1.701	703.03	0.11
271.991	1.705	697.26	0.12
272.996	2.375	697.82	0.11
273.993	3.156	699.47	0.11
274.985	3.933	701.11	0.11
275.979	4.723	702.84	0.11
276.974	5.509	704.53	0.10
277.976	6.282	706.05	0.10
278.983	7.073	707.71	0.10
279.990	7.866	709.37	0.10
279.992	1.837	651.45	0.13
282.002	3.245	654.67	0.12
283.996	4.709	658.35	0.12
285.981	6.161	661.92	0.11
287.998	7.658	665.71	0.11
279.990	2.263	656.09	0.12
280.999	3.004	657.93	0.12
282.000	3.738	659.73	0.12
282.997	4.471	661.53	0.12
283.994	5.206	663.33	0.11
284.991	5.945	665.16	0.11
285.980	6.672	666.91	0.11
286.988	7.415	668.70	0.11
286.989	2.152	612.75	0.14
287.997	2.830	614.63	0.14
289.007	3.515	616.56	0.13
290.010	4.202	618.52	0.13
290.993	4.873	620.43	0.13
291.992	5.554	622.33	0.12
292.992	6.237	624.22	0.12
293.979	6.914	626.12	0.12
294.990	7.606	628.02	0.12
287.994	2.073	605.60	0.14
290.008	3.449	609.83	0.13
291.990	4.793	613.81	0.13
293.976	6.139	617.70	0.12
295.998	7.508	621.58	0.12
294.989	2.260	563.76	0.16
296.001	2.890	565.94	0.15
296.996	3.510	568.06	0.15
298.005	4.136	570.16	0.15
299.004	4.753	572.16	0.14
299.990	5.366	574.18	0.14
300.992	5.994	576.26	0.14
301.980	6.608	578.22	0.13
302.992	7.239	580.26	0.13
303.990	7.856	582.17	0.13
295.996	2.887	565.94	0.15
298.002	4.136	570.18	0.15
299.986	5.374	574.30	0.14
301.976	6.617	578.37	0.13
303.987	7.880	582.48	0.13
305.000	2.547	500.83	0.20
305.998	3.099	503.30	0.19
306.986	3.638	505.61	0.18
307.990	4.193	508.02	0.18
308.979	4.747	510.45	0.17
309.986	5.293	512.64	0.17
310.985	5.841	514.87	0.16
311.987	6.395	517.12	0.16
313.003	6.957	519.38	0.15
314.005	7.514	521.62	0.15
314.998	8.077	523.93	0.15
309.986	3.026	473.87	0.21
310.986	3.546	476.46	0.21
311.986	4.064	478.98	0.20
313.003	4.591	481.48	0.19
314.004	5.108	483.87	0.19
314.997	5.618	486.17	0.18
316.004	6.135	488.45	0.17
316.997	6.648	490.70	0.17
317.988	7.164	492.96	0.17
318.988	7.682	495.18	0.16
319.990	8.204	497.41	0.16
314.995	3.215	440.19	0.25
316.003	3.693	442.86	0.24
316.996	4.167	445.46	0.23
317.989	4.644	448.06	0.22
318.987	5.122	450.61	0.21
319.989	5.602	453.10	0.21
321.007	6.092	455.63	0.20
322.011	6.573	458.04	0.19
323.006	7.052	460.40	0.19
324.000	7.530	462.72	0.18
324.993	8.008	465.01	0.17

a Speed of sound values listed are averaged from up to 12 measurements at each state point; lines separate the isochores. The standard uncertainties for temperature, pressure, and composition are *u*_c_(*T*) = 0.005 K, *u*_c_(*p*) = 0.014 MPa, and 0.003 mole fraction, respectively.

**Table 4:** Experimental Speed of Sound Data for the R-1132(E)/1234yf Mixture with a Composition of 0.435 Mole Fraction R-1132(E) and 0.565 Mole Fraction R-1234yf (*c*) Listed with the Corresponding Temperature (*T*), Pressure (*p*), and Relative Expanded Uncertainty (*U*_r_(*c*)) Determined with a Coverage Factor, *k* = 2^a^

*T*/K	*p*/MPa	*c*/m·s^–1^	100·*U*_r_(*c*)
230.000	0.681	790.04	0.086
235.014	1.008	767.80	0.085
240.007	1.071	743.95	0.085
245.010	1.080	719.76	0.084
250.007	1.188	696.28	0.083
251.996	1.251	687.28	0.083
253.998	2.318	686.31	0.082
254.997	3.127	688.03	0.081
256.013	3.843	689.03	0.080
257.014	4.581	690.18	0.080
258.001	5.334	691.52	0.079
259.004	6.096	692.87	0.078
260.007	6.856	694.20	0.077
260.996	7.615	695.58	0.077
259.987	0.658	642.69	0.083
261.974	1.039	636.34	0.083
263.993	1.115	627.20	0.083
265.988	1.138	617.64	0.083
267.985	1.150	607.95	0.083
269.995	1.167	598.22	0.083
272.004	1.186	588.55	0.084
273.989	1.204	578.91	0.084
275.975	1.217	569.17	0.085
277.974	1.242	559.45	0.085
280.003	1.302	549.95	0.086
282.012	1.409	541.14	0.087
283.990	1.631	533.87	0.087
260.006	1.904	654.19	0.082
260.995	2.623	655.65	0.081
261.994	3.346	657.11	0.080
262.995	4.073	658.60	0.079
264.013	4.819	660.14	0.078
265.014	5.544	661.60	0.078
265.015	5.538	661.55	0.078
266.008	6.226	662.80	0.077
266.998	6.935	664.17	0.076
267.988	7.648	665.58	0.075
267.987	1.957	616.33	0.082
268.996	2.632	617.84	0.081
269.996	3.303	619.36	0.080
271.001	3.977	620.88	0.079
272.004	4.647	622.38	0.078
273.009	5.330	623.96	0.077
274.004	5.990	625.40	0.076
274.998	6.658	626.89	0.076
275.991	7.329	628.41	0.075
276.987	8.001	629.92	0.074
276.988	1.323	565.83	0.085
277.990	1.935	567.50	0.083
278.996	2.550	569.17	0.082
280.004	3.167	570.85	0.081
281.012	3.783	572.51	0.080
282.013	4.397	574.17	0.079
283.009	5.007	575.80	0.078
284.007	5.617	577.41	0.077
285.006	6.229	579.03	0.076
286.008	6.842	580.64	0.075
286.009	6.839	580.62	0.075
287.018	7.457	582.23	0.075
288.010	8.071	583.86	0.074
285.993	2.221	530.94	0.087
288.010	3.170	532.25	0.085
290.007	4.206	534.74	0.083
292.004	5.306	537.90	0.080
293.992	6.397	541.03	0.078
295.999	7.510	544.25	0.076
290.006	2.352	512.77	0.090
292.003	3.347	515.31	0.087
293.993	4.383	518.33	0.085
295.999	5.449	521.57	0.082
298.019	6.530	524.91	0.080
300.003	7.604	528.26	0.078
288.010	1.124	507.54	0.093
289.020	1.287	504.46	0.094
290.008	1.804	506.13	0.092
290.990	2.321	507.73	0.091
291.990	2.921	510.21	0.089
292.989	3.419	511.62	0.088
293.977	3.933	513.25	0.086
294.987	4.517	515.39	0.085
295.998	5.063	517.18	0.084
296.994	5.565	518.66	0.083
298.002	6.103	520.32	0.081
299.002	6.655	522.16	0.080
299.986	7.194	523.97	0.079
301.005	7.704	525.32	0.078
300.003	1.613	451.10	0.112
301.004	2.054	452.69	0.110
301.993	2.514	454.54	0.108
303.005	2.997	456.55	0.106
304.003	3.496	458.77	0.104
305.016	3.981	460.79	0.102
306.013	4.437	462.49	0.100
307.017	4.906	464.26	0.098
308.005	5.374	466.07	0.097
309.010	5.862	468.01	0.095
310.000	6.345	469.95	0.093
311.000	6.822	471.79	0.092
312.017	7.306	473.60	0.090
313.001	7.758	475.19	0.089
301.993	2.529	454.57	0.108
304.002	3.441	457.89	0.104
306.014	4.379	461.48	0.100
308.004	5.311	465.07	0.097
310.002	6.251	468.65	0.094
312.018	7.210	472.29	0.091
309.983	1.721	397.08	0.150
310.999	2.142	399.09	0.146
312.000	2.549	401.37	0.143
313.001	2.963	403.83	0.140
314.019	3.385	405.95	0.136
315.011	3.795	407.97	0.133
316.002	4.208	409.99	0.131
316.994	4.621	411.98	0.128
318.002	5.044	414.02	0.125
319.001	5.463	416.03	0.123
320.002	5.882	417.98	0.120
321.005	6.303	419.91	0.118
322.008	6.723	421.82	0.115
323.004	7.133	423.65	0.113
324.014	7.552	425.49	0.111
309.982	1.713	397.34	0.150
310.997	2.135	399.58	0.146
311.998	2.555	401.76	0.143
312.999	2.971	403.96	0.139
314.017	3.398	406.03	0.136
315.009	3.797	408.01	0.134
316.000	4.201	409.91	0.131
316.992	4.613	411.86	0.128
318.001	5.033	413.86	0.125
319.000	5.455	415.88	0.123
320.002	5.879	417.90	0.120
321.004	6.301	419.87	0.118
322.008	6.719	421.77	0.115
323.004	7.126	423.52	0.113
324.014	7.543	425.33	0.111
325.007	7.953	427.11	0.109
322.009	2.453	341.30	0.223
324.014	3.165	346.17	0.210
326.004	3.864	350.65	0.198
328.008	4.573	354.99	0.188
330.016	5.236	358.42	0.180
332.013	5.938	362.41	0.171
334.003	6.648	366.44	0.163
336.008	7.361	370.36	0.156
338.003	8.068	374.10	0.150
326.002	2.988	329.56	0.241
328.005	3.667	334.60	0.227
330.015	4.345	339.32	0.214
332.011	5.023	343.67	0.202
334.003	5.662	347.17	0.193
336.007	6.336	351.13	0.184
338.003	7.012	355.04	0.175
340.007	7.693	358.89	0.167
341.991	8.365	362.57	0.160
326.006	3.008	330.53	0.239
328.026	3.689	335.18	0.225
330.033	4.366	339.65	0.213
332.013	5.037	343.96	0.202
334.005	5.677	347.52	0.193
336.009	6.346	351.38	0.183
338.021	7.026	355.29	0.175
340.025	7.705	359.10	0.167
341.007	8.039	360.93	0.163
326.004	2.987	329.91	0.241
328.023	3.672	334.80	0.226
330.032	4.353	339.38	0.213
332.011	5.023	343.61	0.202
334.003	5.646	346.80	0.194
336.008	6.325	350.92	0.184
338.020	7.009	354.92	0.175
340.024	7.699	358.89	0.167
341.007	8.037	360.82	0.163

a Speed of sound values listed are averaged from up to 12 measurements at each state point; lines separate the isochores. The standard uncertainties for temperature, pressure, and composition are *u*_c_(*T*) = 0.005 K, *u*_c_(*p*) = 0.014 MPa, and 0.004 mole fraction, respectively.

### 3.2 Comparison to Multifluid Model

**Figure 6:**
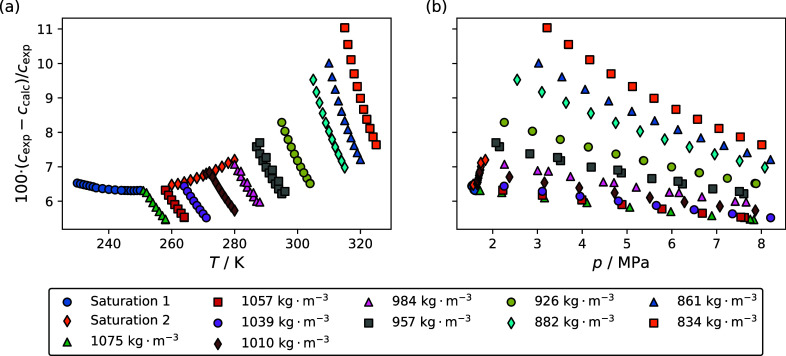
Comparisons of the experimental speed of sound data reported in this study for the R-1132(E)/32 blend, *c*_exp_, to those calculated with a multifluid model incorporating the Helmholtz-energy-explicit equations of state of Akasaka and Lemmon^9^ for R-1132(E) and Tillner-Roth and Yokozeki^10^ for R-32, *c*_calc_. The binary interaction parameters are for the chemically similar system R-32/1234yf reported by Akasaka,^13^ which were suggested by REFPROP version 10.0.^12^ Different symbols correspond to the different isochores investigated.

**Figure 7:**
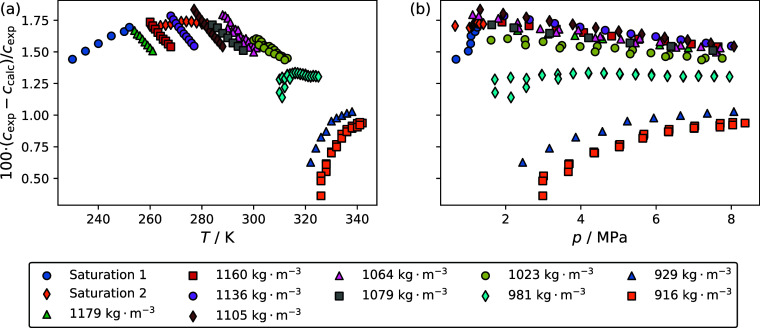
Comparisons of the experimental speed of sound data reported in this study for the R-1132(E)/1234yf blend, *c*_exp_, to those calculated with a multifluid model incorporating the Helmholtz-energy-explicit equations of state of Akasaka and Lemmon^9^ for R-1132(E) and Lemmon and Akasaka^11^ for R-1234yf, *c*_calc_. The binary interaction parameters are for the chemically similar system R-1234yf/1234ze(E) reported by Lemmon,^14^ which were suggested by REFPROP version 10.0.^12^ Different symbols correspond to the different isochores investigated.

Figures 6 and 7 are deviation plots comparing the experimental speed of sound data to those calculated using multifluid models for the R-1132(E)/32 and R-1132(E)/1234yf blends, respectively. Figures 6 and 7 have panels a and b, which show the deviations as a function of temperature and pressure, respectively. The Helmholtz-energy-explicit EOSs incorporated in the multifluid models are the R-1132(E) EOS of Akasaka and Lemmon,^9^ the R-32 EOS of Tillner-Roth and Yokozeki,^10^ and the R-1234yf EOS of Lemmon and Akasaka.^11^ Presently, no studies in the literature report mixture parameters for R-1132(E) blends with R-32 or R-1234yf. Therefore, the mixing parameters used are the default parameters included in REFPROP version 10.0.^12^ This estimation scheme is discussed in more detail by Bell et al.^22^ For the R-1132(E)/32 blend, the parameters used were those reported by Akasaka^13^ for the chemically similar R-32/1234yf blend. For the R-1132(E)/1234yf mixture, the parameters selected by REFPROP version 10.0 were for R-1234yf/1234ze(E) reported by Lemmon.^14^Figure 6 shows that deviations between the experimental speed of sound data and the multifluid model for the R-1132(E)/32 blend range from 5.5 to 11.0%. Figure 7 shows that for the R-1132(E)/1234yf blend, the deviations range from 0.4 to 1.8%. In both cases, the deviations are significantly greater than the uncertainty in the speed of sound measurements.

The overall performance of the predictions is characterized by the absolute average deviation defined by
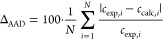
2where *c*_exp,*i*_ is an experimental data point, *c*_calc_,_*i*_ is a speed of sound value calculated using the multifluid model, and *N* is the total number of data points included in the average. For the R-1132(E)/32 blend, the Δ_AAD_ was 7.0%, and for the R-1132(E)/1234yf blend, it was 1.4%. It is not surprising that the models exhibit deviations greater than the reported speed of sound uncertainty because binary interaction parameters for chemically similar systems were used. Additionally, the greater deviations seen for the R-1132(E) blend with R-32 may be the result of known weaknesses in the Helmholtz-energy-explicit EOS for R-32 in accurately representing its speed of sound, as highlighted in our previous study^18^ and that by Bell.^23^ To improve the representation of the speed of sound with these multifluid models, dedicated binary interaction parameters for each system must be fit, and the EOS for R-32 should be improved.

## 4 Conclusions

The speed of sound of binary mixtures containing 0.336 mole fraction *trans*-1,2-difluoroethylene (R-1132(E)) with difluoromethane (R-32) or 0.435 mole fraction R-1132(E) with 2,3,3,3-tetrafluoropropene (R-1234yf) was measured with a dual-path pulse-echo instrument. The speed of sound was measured at temperatures ranging from 230 to a maximum temperature of 323 K for blends with R-32 and 342 K for blends with R-1234yf. Measurements were limited to a maximum pressure of 8 MPa to avoid pressure-driven disproportionation reactions of R-1132(E) reported by Goto et al.^8^ The data were compared to multifluid models incorporating the EOS of Akasaka and Lemmon^9^ for R-1132(E), Tillner-Roth and Yokozeki^10^ for R-32, and Lemmon and Akasaka^11^ for R-1234yf. Binary interaction parameters for chemically similar systems, suggested by REFPROP version 10.0,^12^ were used, which were the parameters for the binary R-32/1234yf reported by Akasaka^13^ for the R-1132(E)/32 blend and the parameters for the binary R-1234yf/1234ze(E) reported by Lemmon^14^ for the R-1132(E)/1234yf blend. Deviations between the multifluid models and the data reported in this study were from 5.5 to 11.0% for the R-1132(E)/32 blend and 0.4 to 1.8% for the R-1132(E)/1234yf blend. These deviations were summarized using Δ_AAD_, which were found to be 7.0% for the R-1132(E)/32 blend and 1.4% for the R-1132(E)/1234yf blend. These deviations were unsurprising given that binary interaction parameters for chemically similar systems were used. The greater deviations for the R-1132(E)/32 blend than the R-1132(E)/1234yf blend were also unsurprising given the weaknesses in the R-32 EOS of Tillner-Roth and Yokozeki^10^ in accurately representing the speed of sound.^18^ To improve the representation of the speed of sound with these multifluid models, dedicated binary interaction parameters for each system must be fit, and the EOS for R-32 should be improved.

The data reported in this study will be used to refit both the R-1132(E) EOS and binary interaction parameters for the R-1132(E)/32 and R-1132(E)/1234yf blends. The instability issues seen with R-1132(E) at high pressures preclude its use as a pure working fluid. However, the more promising application of R-1132(E) is in multicomponent refrigerant blends such as R-474A, which is currently being tested to heat and cool automobiles. Accurate multifluid models for mixtures containing R-1132(E) will enable more realistic cycle simulations to screen for applications of R-1132(E) blends. This research supports the development of a more sustainable, energy-efficient heating and cooling infrastructure, which aims to meet global refrigeration demands while limiting the environmental impact.

## Supporting Information Available

Unaveraged speed of sound data for the R-1132(E)/32 blend and R-1132(E)/1234yf blend (ZIP)

## Notes

Commercial equipment, instruments, or materials are identified only to adequately specify certain procedures. Such identification does not imply recommendation or endorsement by the National Institute of Standards and Technology, nor does it imply that the identified products are necessarily the best available for the purpose. Contribution of the National Institute of Standards and Technology. Not subject to copyright in the United States.
